# Scientific Researches into the Causes of Alcoholism and Inebriety

**Published:** 1916-09

**Authors:** T. D. Crothers

**Affiliations:** Hartford, Conn.


### Scientific Researches Into the Causes of Alcoholism and Inebriety.

T. D. CROTHERS, M. D., Hartford, Conn.

One great fact has been established by accurate laboratory and clinical research, viz., that the physiological action of alcohol on the cell and tissue is that of an anesthetic and depressant, and not a tonic or stimulant. This has been accepted by the profession generally, and while it revolutionizes the previous theories, explains in some degree why alcohol is so fascinating.

Beyond this, there is a vast range of causes producing alcoholism and inebriety that are practically unknown. All remedial and restorative efforts are based on the theory that alcohol is the special and particular cause of all the degenerations which follow from its use.

Careful studies of individual cases show this to be untrue; also that in many instances alchol is only a symptom. It may be a complicating drug intensifying unknown conditions that were latent before. It may be a specific poison localizing in certain organs. It is also cumulative, and associated with the most complex neuroses.

The causes that impel men to drink have never been studied scientifically. The literature up to the present is a confusing mass of theories and opinions unverified.

In this unknown region there are innumerable questions like the following: Why are certain periods of life more favorable for the outbreak of the craze for alcohol than others? Why does the desire to drink break out suddenly in diverse conditions, and then subside from causes inadequate to explain the change? What is the explanation of the exact periodicity of these drink excesses that are as certain as the rise and fall of the tide? What are the causes in surroundings and conditions of living that provoke these paroxysms? Why do men drink after injuries, diseases, shocks, losses, disappointments, business reverses and great successes in life? What degenerations are transmitted from the parents to the children that create susceptibility or immunity to the effects of alcohol? Why are some persons able to drink in so-called moderation for years, and why do others quickly become diseased and die? Why do some men drink in early life, then abstain, and in middle or later life turn to alcohol again and drink until death? Why are some persons susceptible to the contagion of surroundings and companions, while others are immune? What physical and psychical causes produce the drink craze?

These are some of the unknown causes and conditions which have never been studied with scientific exactness. One of the most prominent and widely accepted explanations is the so-called moral cause. Physical conditions are considered results and not causes.

A Research Foundation has recently been organized at Hartford, Conn., for the purpose of making an exact scientific study of these questions. It will be endowed and become a permanent work. Preliminary studies have already begun, and practicing physicians from all parts of the country are appealed to for the records and histories of cases which will be compiled and tabulated for the purpose of determining the laws which control and govern them.

This is the first scientific effort to take up the subjects of alcoholism and inebriety and determine the causes which produce them outside of alcohol. Science has shown that these conditions are governed by exact physical and psychical laws, which if known and understood would indicate the most practical means and measures of relief.

The Foundation will be practically a laboratory or clearinghouse where persons can come for examination, counsel and advice. To a large class of persons who want something more than pledges, appeals or sanatorium treatment, this will open a new field of means and measures for relief that will be most welcome.

Correspondence is earnestly solicited from the profession.

---

**Superfoetation.** A 12-para negress in 1900 stated that she had menstruated twice after she was sure she was pregnant. A seven-month male foetus weighing 5 pounds and a nine-month foetus apparently fully matured were delivered at one labor. Both were "raised" and are alive at present, with about 20 pounds difference in weight. Onslow Regan, Alexander City, Ala., Med. World, Aug.

---

**Chlorophyl.** Burgi, Correspondenzblatt fur Schweizer Aertzte, Apr. 16, ascribes a direct haematopoietic function to chlorophyl, rather than assuming that the generally conceded value of spinach, etc., in anaemia is due to a large amount of iron. Maillart, ibid. June 3, corroborates this view from the historic standpoint, although limiting his discussion to rather narrow geographic regions. (Edit., Med. Rec., July 29).

---

**Gas Bacillus Infection.** Franz states that this occurred in 2% of soldiers wounded in Belgium, on account of the highly fertilized soil, the mortality being 53.4%.

